# Supplementary material for: Learning curve and surgical outcome of robotic assisted colorectal surgery with ERAS program
Source: Sci Rep. 2022 Nov 29;12:20566. doi: 10.1038/s41598-022-24665-w (PMC9709162; doi:10.1038/s41598-022-24665-w)
Supplement: Supplementary file 1 — Supplementary Information 1. [file 41598_2022_24665_MOESM1_ESM.zip › LSC Raw data-Ñ[▒K/070/O_ñw╝╨░O▒Kñσ.pdf]

正式摘要(電子簽章：000000186079972)

W83 / W83

臺中榮民總醫院

Operation Note

[redacted]

- 手術日期：2020/02/20
- 手術醫師：VS 蔣鋒帆，CR 江世偉，R 邱俊凱，R 陳思如
- Anesthesia：GE

Diagnosis

- Pre-operative diagnosis : Malignant tumor of rectum FOR LAP CAA
- Post-operative diagnosis : Ditto.

Surgical Indication

- For curative resection

Operation

- 1. Laparoscopic LAR with side-to-end anastomosis, double stapling, GIA 60/4.1mm, CDH 29mm. Re-inforcement of silk: nil, air tight test : done
  2. Bilateral donuts: intact
  3. Re-peritonealization : none
  4. Takedown of splenic flexure: none
  5. Proximal margin: 15cm, distal margin: at least 1cm
  6. Vessel ligation: IMA above left colic artery
  7. No.12 penrose drain x 1 at the pelvis

Finding

- 1. Post per-anal excision scar with central necrosis tissue, about 3 cm x 1 cm in size, located at lower rectum, which invaded to the muscular layer but didn't penetrate through the serosa. No palpable lymph nodes at mesocolon, along intermediate vessel to IMA root. Clinical stage: T2-3N0M0

Procedure

- Under general anesthesia, the patient was placed in Trendelenburg lithotomy position. The abdomen and perineum was prepped and draped in standard sterile fashion. A small incision was made near umbilicus and varess needle was inserted. Pneumoperitoneum was established with CO2 inflation. A 12mm trochar was inserted right lateral of umbilicus. Laparoscopy was inserted and then another 5mm and 12mm trochars were inserted over RUQ and RLQ respectively under laparoscopic guide. Another two 5mm trochar was inserted at LLQ and LUQ under laparoscopic guide. Mesosigmoid colon was hold by grasper and dissection was made to separate mesocolon from retroperitoneum and further dissection was done toward mesorectum. IMA was identified and endoclips were applied. IMA was divided below left colic artery. IMV was also divided after endoclips applied. Sigmoid colon was hold and dissection was made to separate sigmoid colon from peritoneum. The whole sigmoid colon and upper rectum were mobilized. The peritoneum over the selected distal cut end was opened. Laparoscopy and all trochars were removed. A small transverse incision was made at paraumbilical region. The rectum was pulled out and looped by gauze. Vessels of mesorectum were ligated and divdied. Distal irrigation via anus was done. The rectum was divided by GIA 60/4.1mm. The proximal limb was pulled out and dissection was done at the selected site of proximal cut end with ligation of marginal artery.EA avil was applied and the specimen was removed. Side-to-end anastomosis was made by CDH 29mm. One No.12 penrose drain was placed at pelvis. The transverse wound was closed with No.1 Vicryl suture. The wound was irrigated and closed with 3-0 Nylon. Under stable condition, the patient was sent to POR.
- Estimated blood loss: minimum

- 器械數量: 正確
- 紗布數量: 正確

Image

- PAINT
  - abdomen

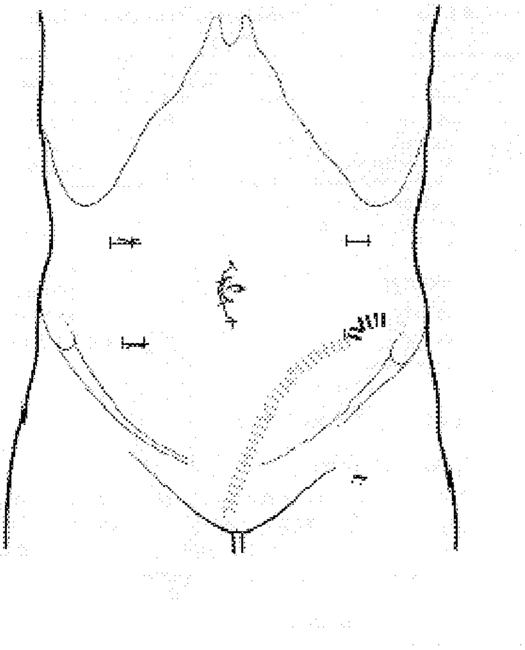

  - colon & rectum

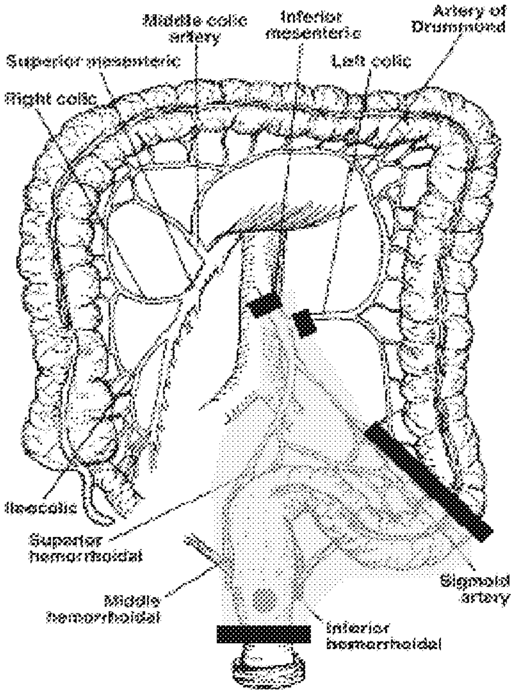

編輯主任 2062F蔣鋒帆、主治 2288K江世偉
正式摘要(電子簽章：000000186079972)

W83 / W83

doctorRank:B
